# Virtual Intervention for Caregivers of Persons With Lewy Body Dementia: Pilot Quasi-Experimental Single-Arm Study

**DOI:** 10.2196/37108

**Published:** 2022-07-29

**Authors:** Oleg Zaslavsky, Jasmine Kaneshiro, Frances Chu, Andrew Teng, Kimiko Domoto-Reilly, Annie T Chen

**Affiliations:** 1 University of Washington Seattle, WA United States

**Keywords:** dementia, caregiver, internet based, digital health, digital intervention, eHealth, feasibility, web-based, peer support, didactic training, caregiving, informal care, spousal care, remote intervention, Lewy body, Lewy bodies, discussion forum, discussion platform, online support, distress, stress reduction, online discussion, support group, discussion group, burden, depression, depressive symptom, lonely, loneliness, mental health

## Abstract

**Background:**

Compared to other types of dementia, family caregivers of people with Lewy body dementia (LBD) report higher stress levels and more severe depressive symptoms. Although several digital support interventions for caregivers of persons with dementia exist, few target LBD specifically or leverage a fully remote and asynchronous approach suitable for pandemic circumstances.

**Objective:**

We performed a pilot evaluation of a digital intervention designed to help caregivers of people with LBD address challenges they have experienced, with the end goal of reducing psychological distress in this population.

**Methods:**

We recruited 15 family caregivers of people with LBD to participate in the quasi-experimental, single-arm, mixed methods study titled Virtual Online Communities for Aging Life Experience–Lewy Body Dementia (VOCALE-LBD). The study offers an 8-week web-based intervention that uses a digital discussion platform and involves moderation, peer-to-peer support, didactic training, and problem-solving skill enactment.

**Results:**

Participants’ baseline characteristics were the following: mean age 66 (SD 8) years; 14 of 15 (93%) of them were female; all (15/15, 100%) were White; and 8 (53%) of them had at least a postgraduate degree. Throughout the intervention, participants engaged in weekly web-based discussions, generating a total of 434 posts (average 4 posts per week). Attrition was 20% (3/15). Upon study exit, participants showed the following average improvements: 3.0 (SD 6.0) in depression, 8.3 (SD 16.7) in burden, 2.9 (SD 6.8) in stress, and 0.3 (SD 0.8) in loneliness. When looking at the proportion of participants with clinically signiﬁcant improvement versus those with a worsening of ≥0.5 SD for each outcome, we observed net improvements of 50% (6/12), 33% (4/12), 25% (3/12), and 25% (3/12) in depression, loneliness, burden, and stress, respectively. In terms of the benefits of participation, participants reported that participation helped them “a great deal” to (1) improve their understanding of LBD (9/12, 75%), (2) gain confidence in dealing with difficult behaviors of the care recipient (6/12, 50%), and (3) improve in one’s abilities to provide care to the care recipient (4/12, 33%).

**Conclusions:**

The study generated promising feasibility and preliminary efficacy data for a low-cost, web-based intervention designed for caregivers of persons with LBD. Though the study was not powered for significance, we observed nominal average and net improvements in important psychological outcomes. Moreover, many caregivers reported that study participation helped them better understand the disease, feel more conﬁdent in dealing with difficult behaviors of the care recipient, and improve their ability to care for the care recipient. If validated in future studies, the intervention could be an accessible, on-demand resource for caregivers, enabling them to engage in moderated remote discussions with peers at their own convenience in terms of location, time of the day, and frequency.

## Introduction

Lewy body dementias (LBDs), referring to both dementia with Lewy bodies and Parkinson disease dementia (PDD), are the second most common type of degenerative dementia in older adults [1]. These are complex disorders in which patients often exhibit disruptive behaviors that make caregiving challenging [1,2]. Compared to other types of dementia, caregivers of people with LBD report higher stress [3] and more severe depressive symptoms [4]. Many were unsatisfied with the support they received, even in pre–COVID-19 contexts [5]. The ongoing COVID-19 pandemic has multiplied the challenges for family caregivers of persons with LBD [6]. First, changes in daily life, such as limited in-person social contact, can exacerbate neurobehavioral symptoms, causing significant distress to caregivers [7]. Second, as older persons with dementia are especially susceptible to COVID-19 complications [8], caregivers of persons with LBD may engage in more restrictive, prolonged protective practices that affect their own opportunities for peer-to-peer connection, socialization, and physical and cognitive stimulation. Finally, many caregivers rely on support services that could be severely disrupted or even permanently discontinued amid the ongoing pandemic. As such, support interventions that conform to “the new normal” realities in the COVID-19 era for caregivers of persons with LBD are warranted.

A proliferation of interventions for informal caregivers of persons living with dementia has occurred in recent years, including digital interventions focusing on caregiver empowerment and the psychological health [9-13]. However, many of the digital interventions do not account for the unique characteristics of their target users, affecting their effectiveness and implementation potential [14], and still include in-person elements that might be suboptimal in pandemic circumstances. Many lack a fully remote, asynchronous approach that allows on-demand engagement in terms of location, time, and frequency and do not focus on LBD specifically. A fully remote, asynchronous intervention specifically designed for family caregivers of persons living with LBD, leveraging peer-to-peer support, may fill the gap and improve psychological health in this population.

This study examined the feasibility of such an intervention, called Virtual Online Communities for Aging Life Experience–Lewy Body Dementia (VOCALE-LBD), in terms of recruitment, retention, and preliminary efficacy concerning the following psychological outcomes: caregiver burden, depressive symptoms, stress, and loneliness using a quasi-experimental, single-arm, mixed methods design. Owing to the preliminary nature of the study, the quantitative analyses were not powered for significance but were used to examine whether the intervention was associated with trends in the expected directions of the outcome measures. The qualitative data, on the other hand, provided opportunities to better understand participants’ experiences with the intervention and areas for improvement.

## Methods

### Study Design

This was a prospective, one-group, pre-post study to assess the feasibility and preliminary efficacy of a new web-based intervention involving moderation, peer-to-peer support, didactic training, and problem-solving skill enactment. Participants were granted access to the VOCALE-LBD intervention hosted on a private website.

### Ethical Considerations

The University of Washington’s (UW’s) institutional review board (IRB) approved the study protocol (approval 13431). At enrollment, participants provided informed consent to be included in the study.

### Participants

Participants were recruited from the Memory and Brain Wellness Center (MBWC) at Harborview Medical Center in Seattle, Washington. The MBWC encompasses both the MBWC clinic and the Alzheimer’s Disease Research Center (ADRC) and has been designated as a Lewy Body Dementia Association Research Center of Excellence [15]. The MBWC evaluates over 1000 new patients on an annual basis, and it is the only major academic medical center serving the 5-state region of Washington, Wyoming, Alaska, Montana, and Idaho. The ADRC maintains a continually updated, comprehensive contact list of persons who have signed an IRB-approved consent form to be contacted about participation in research studies conducted by UW-affiliated researchers. Many of the Research Registry members have been evaluated at the MBWC clinic and hence have a recent, reliable clinical diagnosis of specific types of dementia such as Alzheimer disease, Parkinson disease dementia (PDD), Dementia with Lewy bodies (DLB), etc. Family members of clinic patients are also eligible to join the registry. All have expressed willingness to be contacted for potential study participation. Eligibility criteria for this study were as follows: being family or informal caregiver of a person with a diagnosis of LBD; being able to read, write, and speak English; having a device that can access the internet and be used for videoconferencing or telephone calls; and being ≥18 years old. Of note, when possible, MBWC clinicians aim to distinguish between DLB and other subtypes of dementia, such as PDD, and to educate patients and their caregivers on the exact diagnosis. As such, most, if not all, study participants cared for people who were highly likely to have a diagnosis of DLB. Participants were compensated up to US $250 depending on their participation.

### Intervention

**Figure 1:**
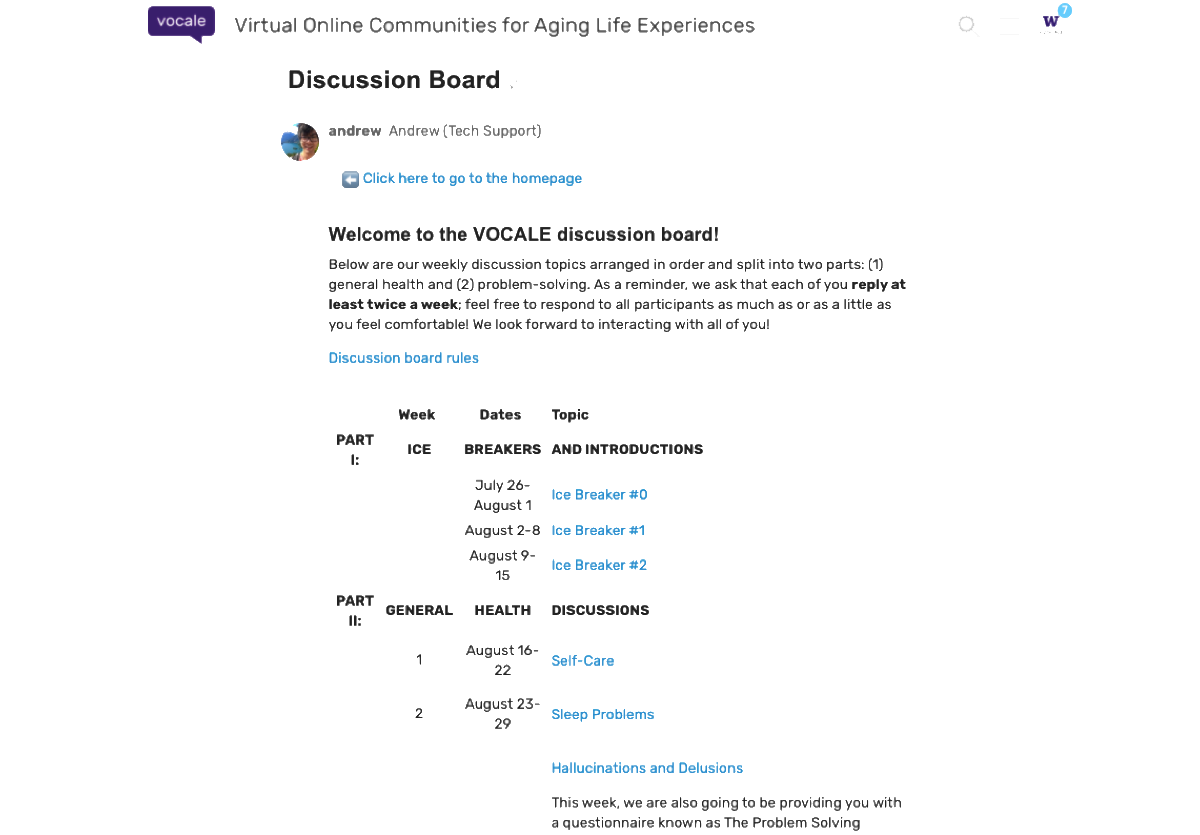
Landing page of Virtual Online Communities for Aging Life Experience (VOCALE)–Lewy Body Dementia.

**Figure 2:**
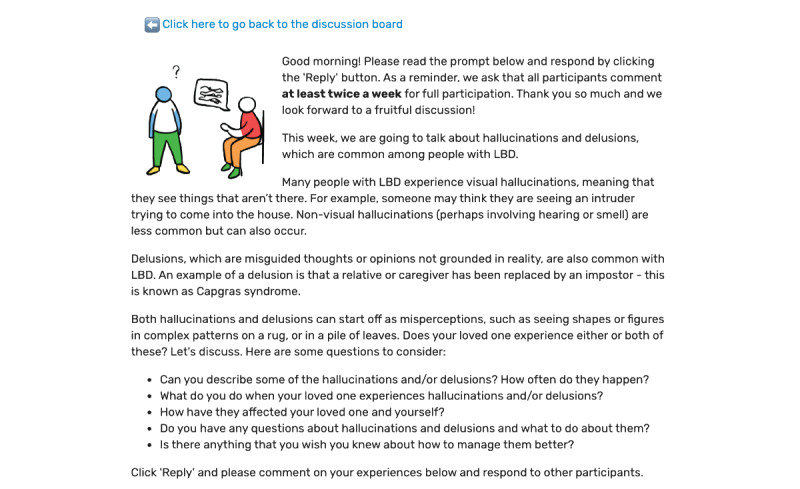
Virtual Online Communities for Aging Life Experience–Lewy Body Dementia sample weekly discussion prompt. LBD: Lewy body dementia.

We adapted a prior social networking intervention that was developed for older adults with prefrailty and frailty, VOCALE [16-18], to the needs of caregivers of individuals with LBD. Similar to our previous studies, the refined intervention included training and moderated web-based discussion components. Training sessions were performed remotely; participants were introduced to the platform (Figure 1) and described activities to learn how to interact with the discussion board. Weekly thematic discussion prompts (Figure 2 shows an example of a prompt concerning hallucinations) allowed participants to respond to a specific topic of interest at their leisure and provided the participants an opportunity to interact with each other. The first 3 weeks were focused on the most salient LBD caregiving experiences featured in previous literature [5,19,20] and our previous formative work. Briefly, in this formative work, we conducted 8 individual interviews and 2 focus groups with caregivers of persons with LBD to identify relevant topics for the intervention. We used open-ended prompts to elicit ideas that caregivers would be interested in discussing during the study. Sleep problems, hallucinations and delusions, and self-care emerged as the topics of greatest interest and thus became our topics for the first 3 weeks.

**Figure 3:**
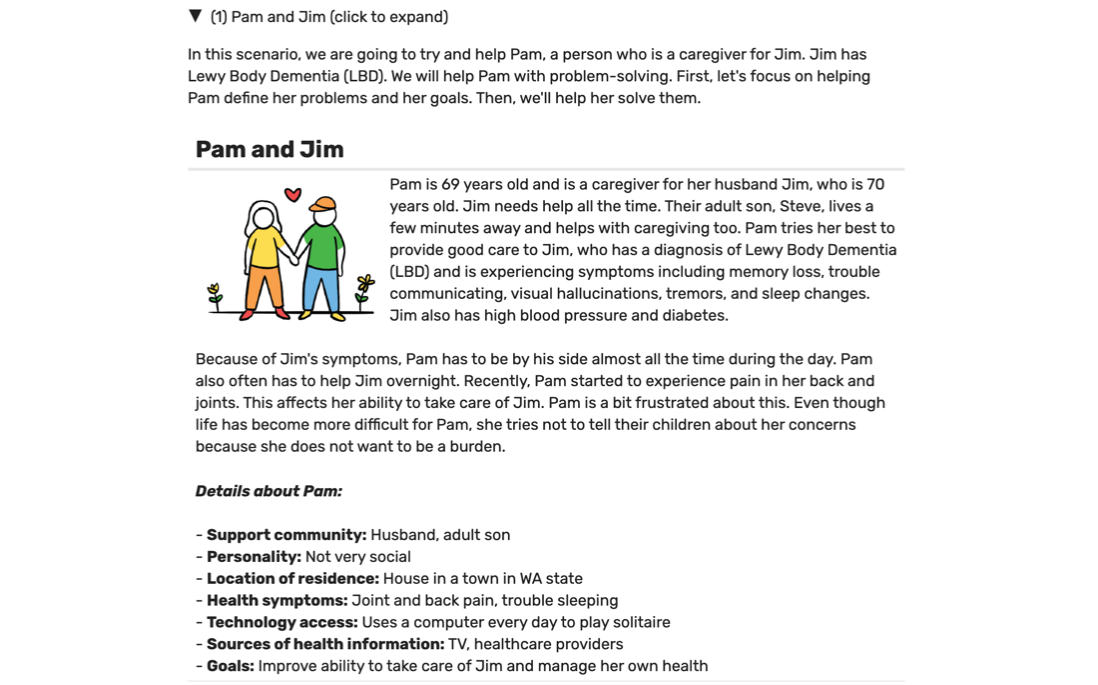
Virtual Online Communities for Aging Life Experience–Lewy Body Dementia persona page.

The next 5 weeks involved psychoeducational materials based on problem-solving therapy (PST), a cognitive behavioral intervention focused on the adoption and application of adaptive problem-solving attitudes and skills [21]. PST has been used with individuals with different types of problems, including caregivers [22,23] and older adults with other health issues [24]. The intervention also incorporated personas or prototypical examples of caregivers of persons with LBD (Figure 3 shows an example of a persona). Personas, or representations or archetypes grounded in real data, are often used in user-centered design to help inform product design [25]. In this context, we used personas to enable participants to practice solving realistic problems that they might face. Working with the personas also provided some other potential benefits, such as being able to practice problem-solving without sharing anything too personal, as well as having an outlet to focus on someone besides themselves.

Two research staff members served as the study’s moderators. Both were advanced practice nurses who received moderator training from the study team. Moderators logged in at least 3 times each weekday and at least once per weekend day to review any new comments and add comments to address questions, provide emotional support and validation, encourage dialogue, and redirect discussions as needed to stay on topic. The moderators did not provide any medical advice. Moderators also monitored for comments with inaccurate information and addressed them as needed through private email or discussion board comments. Lastly, moderators sent personalized reminder emails once a week to participants who had not yet posted. They replied to most participants’ comments.

### Data Collection Procedures

All data collection sessions in this pilot were virtual and took place on Zoom. The study team collected data using Research Electronic Data Capture (REDCap) [26], a web-based survey platform, at 2 time points: baseline and post intervention. At baseline, the research coordinator provided training on the discussion board website and administered a set of demographic and clinical questionnaires (described in *Measures*). The second postintervention assessment occurred between weeks 9 and 10 and included another set of questionnaires and exit interviews. Exit interviews lasted approximately an hour each and were conducted with a semistructured interview guide that asked for caregiver feedback on topics including various program components, whether people felt a sense of community or support, what was learned or gained from the discussion, and motivations to participate. Caregivers were also given the opportunity to provide any additional feedback or suggestions.

### Measures

#### Depressive Symptoms

We used the 20-item version of the Center for Epidemiologic Studies Depression (CES-D) Scale to assess depression [27]. For each statement, respondents indicated how often they felt depressed during the past week using a scale from 0=rarely or never to 3=most or all of the time. Scores could range from 0 through 60, with higher scores indicating an increased presence of depression symptoms.

#### Caregiver Burden

We used the full 22-item version of the Zarit Caregiver Burden Interview [28]. Caregivers rated each item on a 5-point scale from 0=never to 4=nearly always, yielding a possible range of 0 to 88. Higher values indicated greater levels of caregiver burden.

#### Perceived Stress

We used the full 10-item version of the Perceived Stress Scale [29]. Caregivers rated each item on a 5-point scale from 0=never to 4=very often, yielding a possible range of 0 to 40. Higher values indicated greater perceived stress.

#### Loneliness

We used a short 3-item version of the Revised UCLA Loneliness Scale [30]. Its possible range was from 0 to 6 with higher scores indicating greater loneliness.

#### Social Support

We used a 9-item questionnaire from the Medical Outcomes Study [31]. The questionnaire was designed to assess the amount of social support the participant had available. Responses were scored on a 5-point scale ranging from “none of the time” to “all of the time.” We computed total social support scores by summing the scores for all items after recoding responses. Scores could range from 0 through 45, with higher scores indicating increased social support.

#### Self-efficacy

We used a 5-item Health Self-Efficacy Measure [32]. For each statement, respondents indicated their level of agreement concerning their health management using a scale from 0=strongly disagree to 4=strongly agree. Scores could range from 0 through 20, with higher scores indicating stronger health self-efficacy.

#### Benefits of Participation

On conclusion of the study, we asked all caregivers 4 questions adapted from the REACH II study [33] about ways in which they beneﬁted from participating in the study. Specifically, we asked whether their participation helped improve their understanding of LBD, improve their confidence and ability to deal with difficult behaviors of the care recipient, and make their caregiving life easier. The response options for each question were “not at all,” “some,” and “a great deal.”

### Data Analysis

Descriptive statistics summarized demographics at baseline and outcome distributions by measurement occasions. We also calculated mean (SD) pre- and postintervention change scores for each of the measurements. Next, for each outcome, we calculated the number and percentage of individuals who improved by at least 0.5 SD from baseline to post intervention, and the number and percentage of those who worsened by at least 0.5 SD, and then we subtracted the former form the latter to calculate a net improvement score. We also calculated the number and percentage of individuals selecting response options from the benefit of the participation questionnaire.

Exit interviews were recorded and transcribed verbatim. We analyzed the data using an inductive method adapted from previous research concerning digital interventions for behavioral change [34]. The research team reviewed transcripts line by line and then created a table to organize interview data at the participant level into categories. Categories were based on the interview guide, such as motivations to participate in the study, what was learned from the experience, and participants’ thoughts on different program components. Suggestions, critiques, and other comments from participants were categorized as actionable, not actionable, or needing further discussion and consideration in the future. After each participant’s interview, data were added to the table, and common responses were tabulated. Data abstraction was performed by JK, and all the coauthors participated in the review of the table.

## Results

### Participants

**Figure 4:**
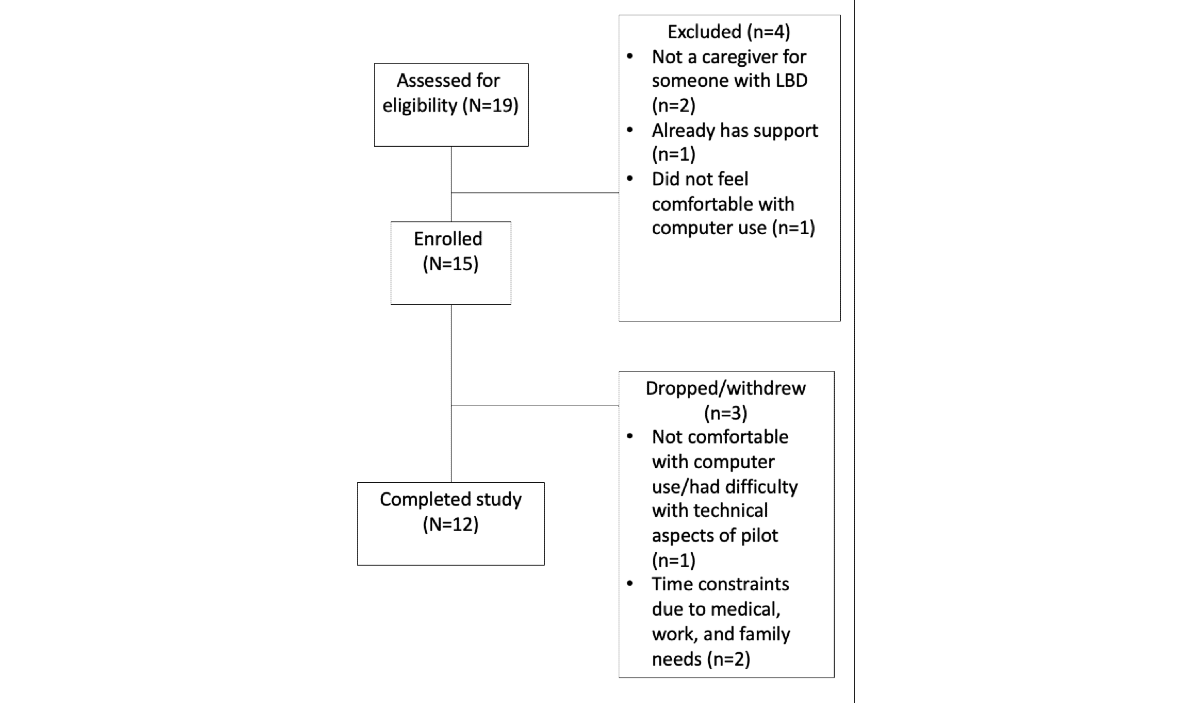
Flowchart for study screening, enrollment, and completion. LBD: Lewy body dementia.

Of the 19 potential candidates screened, 15 were eligible, consented to the study, and provided baseline data (Figure 4). A total of 3 participants left the study at weeks 2, 3, and 5, which yielded a total of 12 participants who completed the full protocol, resulting in an 80% retention rate. Table 1 summarizes the baseline data. The mean participant age was 66 (SD 8) years, all participants (n=15, 100%) were White, and 93% (14/15) were female. In total, 8 (53%) participants had at least a postgraduate degree, and 80% (12/15) of the participants provided more than 40 hours of care per week.

**Table 1:** Participant demographics (baseline; N=15).

Variables			Values
**Sex, n (%)**			
	Female	14 (93)	
	Male	1 (7)	
Age (years), mean (SD)			65.8 (8.3)
Caregiver relationship: spouse or partner, n (%)			15 (100)
White, not of Hispanic/Latino ethnicity, n (%)			15 (100)
**Education level, n (%)**			
	High school diploma or General Educational Development	3 (20)	
	Vocational or associate’s degree	3 (20)	
	Baccalaureate degree	1 (7)	
	Master’s degree	5 (33)	
	Doctoral degree	3 (20)	
Time spent as a caregiver (years), mean (SD)			3.8 (2.5)
**Average care time per week, n (%)**			
	Up to 8 hours	1 (7)	
	20-39 hours	2 (17)	
	≥40 hours	12 (80)	
**Computer comfort level, n (%)**			
	Very uncomfortable	2 (17)	
	Neutral	1 (7)	
	Somewhat comfortable	6 (40)	
	Very comfortable	6 (40)	

### Data, Measures, and Analysis

Table 2 summarizes engagement metrics over time. Throughout the intervention, participants engaged in weekly web-based discussions, generating a total of 434 posts (average of 4 posts per week). On study exit (Table 3), participants showed the following mean improvements: 3.0 (SD 6.0) in depression, 8.3 (SD 16.7) in burden, 2.9 (SD 6.8) in stress, and 0.3 (SD 0.8) in loneliness. When we calculated differences in the proportion of participants with clinically signiﬁcant improvement versus those with a worsening of ≥0.5 SD for each outcome (Table 4), we observed net improvements of 50% (6/12), 35% (4/12), 25% (3/12), and 25% (3/12) in depression, loneliness, burden, and stress, respectively. When we assessed the benefits of participation (Table 5), 75% (9/12) of the participants reported that participation helped them “a great deal” to improve their understanding of LBD compared to 25% (3/12) of them selecting the “some” response option. Similarly, in response to the question about confidence in dealing with difficult behaviors of the care recipient, 50% (6/12) of the participants selected “a great deal,” 42% (5/12) of them selected “some,” and 8% (1/12) of them selected “not at all.” Finally, in response to a question about improvement in one’s abilities to provide care to the care recipient, 33% (4/12) of the participants selected “a great deal,” 58% (7/12) of them selected “some,” and 8% (1/12) of them selected “not at all.”

Qualitative data from the exit interviews showed that of the 12 participants who completed the pilot, 92% (11/12) of them reported they had a positive experience. All participants noted that they felt supported by other participants. Motivations for participating in the study included the following: wanting to support others (4/12, 33%), wanting to interact with others in similar situations and obtain feedback (6/12, 50%), and contributing to research (4/12, 33%).

When asked what they learned or gained from the pilot, 50% (6/12) of the participants mentioned the opportunity to hear from others who were caregivers of people with LBD, and 83% (10/12) of them discussed learning from others, such as solutions and strategies that others have used. Two-thirds (8/12, 67%) of participants felt that the problem-solving elements of the pilot were helpful. One-fourth (3/12, 25%) of participants felt that their baseline problem-solving skills were already robust; therefore, the intervention did not contribute to improvement. One-third (4/12, 33%) of participants did not feel that the intervention’s problem-solving framework was helpful, with 2 participants (17%) mentioning that they felt the framework was difficult to apply in real life. All participants offered constructive feedback regarding the personas. For example, one-third (4/12, 33%) of participants felt that they could not relate to one or both personas, and one-third (4/12, 33%) of them felt that the personas needed to be fleshed out with more details to be useful. Over half (7/12, 58%) of the participants would have preferred to discuss participants’ actual problems rather than those of the personas.

**Table 2:** Participant engagement (posts per week).

Participant ID	Posts, n									Total posts, n
	Week 1	Week 2	Week 3	Week 4	Week 5	Week 6	Week 7	Week 8		
1	3	4	2	4	7	7	5	5	37	
2	9	3	2	3	3	6	1	2	29	
3	2	5	2	2	2	4	2	3	22	
4	2	3	2	2	3	1	0	1	14	
5	4	7	3	1	3	0	0	0	18	
6	8	5	15	3	1	3	3	6	44	
7	4	2	0	0	0	0	0	0	6	
8	15	12	8	2	8	10	2	7	64	
9	4	6	5	2	4	3	3	2	29	
10	3	3	4	2	4	2	2	2	22	
11	6	12	5	5	8	10	6	5	57	
12	4	5	5	2	4	2	3	4	29	
13	3	6	9	4	2	8	2	5	39	
14	0	0	0	0	0	0	0	0	0	
15	3	3	2	3	3	3	3	4	24	
Weekly total	70	76	64	35	52	59	32	46	434	

**Table 3:** Baseline, postintervention, and change scores (N=15).

Parameters	Baseline, mean (SD)	Postintervention (n=12), mean (SD)	Change (n=12), mean (SD)
Depressive symptoms	13.3 (9.0)	10.9 (9.9)	–3.0 (6.0)
Health self-efficacy	21.5 (3.3)	21.8 (3.6)	–0.6 (4.3)
Perceived stress	16.2 (7.2)	13.0 (9.0)	–2.9 (6.8)
Loneliness	5.4 (1.7)	5.0 (1.7)	–0.33 (0.8)
Social support	32.3 (7.9)	33.08 (7.1)	–0.33 (4.2)
Caregiving burden	39.1 (10.8)	36.3 (17.0)	–8.3 (16.6)

**Table 4:** Clinically significant changes^a^ (N=12).

Variable	Improved, n (%)	Worsened, n (%)	Net improvement^b^, n (%)
Depressive symptoms	8 (70)	2 (17)	6 (50)
Health self-efficacy	3 (25)	4 (33)	–1 (–8)
Perceived stress	5 (42)	2 (17)	3 (25)
Loneliness	4 (33)	0 (0)	4 (33)
Social support	3 (25)	2 (12)	1 (8)
Caregiving burden	5 (42)	2 (17)	3 (25)

^a^Clinically signiﬁcant improvement and worsening was deﬁned as an unadjusted standardized change of ≥0.5 SD from baseline to follow-up.

^b^Net improvement = participants who improved – participants who worsened.

**Table 5:** Benefits of participating in the study (N=12).

Question or response categories		Participants, n (%)
**1. Did your participation in the study help you better understand Lewy Body Dementia?**		
	Not at all	0 (0)
	Some	3 (25)
	A great deal	9 (75)
**2. Did your participation in the study help you feel more confident in dealing with difficult behavior of the care recipient?**		
	Not at all	1 (8)
	Some	5 (42)
	A great deal	6 (50)
**3. Did your participation in the study help make your life easier?**		
	Not at all	2 (17)
	Some	7 (58)
	A great deal	3 (25)
**4. Did your participation in the study help your ability to care for care recipient?**		
	Not at all	1 (8)
	Some	7 (58)
	A great deal	4 (33)

## Discussion

### Principal Findings

The study generated promising preliminary feasibility and efficacy data for a fully remote intervention designed specifically for family caregivers of persons with LBD. Enrollment and retention were successful, with the study experiencing only a 20% dropout rate. Our study showed retention rates similar to or better than those reported in other recent studies of digital interventions for family caregivers of persons with dementia [14]. A pooled estimate from recent studies that mainly recruited spousal caregivers [9-13] reported an average 66% retention rate, compared to the 80% retention rate reported in this study. Moreover, in the exit interviews, participants confirmed that the experience of participating in the study was valuable and motivated them to continue participating. As the study was not powered for statistical significance, we observed nominal average and net improvements in important psychological outcomes. Additionally, many caregivers reported that study participation helped them better understand the disease, feel more conﬁdent in dealing with difficult behaviors of the care recipient, and improve their ability to care for the care recipient. These encouraging results concerning improvement in psychological measures are on par with those of the hallmark REACH II trial [35], which, according to the recent AHRQ [36] and NASEM [37] reports, meets the threshold for an evidence-based intervention for family caregivers of a person with dementia. Given that VOCALE-LBD and the REACH II protocol share similar elements, such as the provision of information, didactic instruction, role-playing, problem-solving, and skills training, in this era of increased demand for fully remote interventions, this pilot program seems well suited for further research and evaluation.

In this pilot study, we found nominal average and net improvements in important psychological outcomes, similar to changes observed in the hallmark REACH II trial. Specifically using the same approach to calculate clinically significant net improvements, the REACH II trial reported an approximately 30% improvement in depression and 10% in burden in a comparable intervention subgroup in terms of race and ethnicity. Moreover, in terms of benefits of participation, 58% of the participants in the White/Caucasian REACH II intervention subgroup asserted that the intervention helped them understand memory loss a great deal, and similarly, 59% of them asserted that the intervention helped them to some extent to feel more confident in dealing with the care recipient. Our results of 50% and 30% net improvement in depression and burden, respectively, and 75% and 50% endorsements in benefits for participation fall within the upper bound of the REACH II results. As such, pending further research, one cautious interpretation is that the VOCALE-LBD remote intervention for family caregivers of persons with LBD holds promise for further development and evaluations.

Despite the full-time caregiving demands of our study participants, all but 3 participants completed the 8-week intervention (9/12, 80%). The platform is designed to promote convenience and accessibility, allowing the user to engage within the weekly interval at times of day and frequencies based on their own preferences. We are aware of only 5 web-based intervention studies since 2015 in the dementia caregiving context that recruited mostly spousal caregivers. A study by Pot et al [9] included approximately 60% spousal caregivers and had a 44% retention rate. A study by Gustafson et al [10] included approximately 90% spousal caregivers and had an 84% retention rate. A study by Boots et al [11] included approximately 98% spousal caregivers and had a 61% retention rate. A study by Blom et al [12] included approximately 58% spousal caregivers and had a 70% retention rate. A study by Griffiths et al [13] included approximately 73% spousal caregivers and had a 73% retention rate. There may be various factors accounting for the 80% retention rate and engagement throughout this study. At the outset, because participants were recruited from a study registry, they are likely to demonstrate greater motivation and desire to participate. However, participants also commented that they appreciated the sense of community and connection among their peers, as well as the local context of the intervention (all participants were from the Pacific Northwest, with almost all from Washington state). On several occasions, participants discussed local resources and programs and even suggested plans to meet after the end of the study.

### Limitations

This study has some limitations, which also suggest potential future directions. The effects of the intervention, although promising, must be interpreted with caution as the study was not powered for significance and did not include a control group. Moreover, the study focused on immediate health benefits without evaluating whether the changes were sustained over time. Eligible participants were part of a research registry and needed to have access to the internet. Although today, almost 70% of older adults have access to the internet [38], those who do not may respond differently to the intervention. A lack of racial or ethnic and gender diversity is a notable limitation, and there is a need to explore how caregivers of persons with LBD with other racial or ethnic and gender characteristics might experience VOCALE-LBD. Some participants had trouble relating to the personas and expressed a desire for skill enactment to be more focused on their real-life experiences, suggesting a need in future work to incorporate skill enactment activities more into participants’ own lives. Finally, this study was subject to the common limitations of research involving self-report.

### Conclusions

In this study, we performed a pilot evaluation of an innovative digital intervention that empowers caregivers of persons with LBD by providing a support network and enabling them to develop and improve problem-solving skills that they can use for daily challenges. If validated in future studies, the intervention could be an accessible, on-demand resource for caregivers to engage in moderated remote discussions with their peers at their own convenience in terms of location, time of the day, and frequency. The intervention could be used in conjunction with caregiver usual care and as a stand-alone module in circumstances, such as current and future pandemic emergencies when routine professional interventions might not be readily available**.**

## Abbreviations

ADRC: Alzheimer’s Disease Research Center
CES-D: Center for Epidemiologic Studies Depression
DLB: dementia with Lewy bodies
IRB: institutional review board
LBD: Lewy body dementia
MBWC: Memory and Brain Wellness Center
PDD: Parkinson disease dementia
PST: problem-solving therapy
REDCap: Research Electronic Data Capture
UW: University of Washington
VOCALE-LBD: Virtual Online Communities for Aging Life Experience–Lewy Body Dementia
